# Three-stage pattern of rapid increase, plateau, and subsequent decline in vitamin D concentration during pregnancy among Chinese women: a large-scale survey

**DOI:** 10.3389/fnut.2023.1238389

**Published:** 2023-10-16

**Authors:** Huabin Wang, Feifang Zhang, Bingxian Li, Miao Fu, Xiaoyun Shan, Yongjun Ma

**Affiliations:** ^1^Department of Clinical Laboratory, Affiliated Jinhua Hospital, Zhejiang University School of Medicine, Jinhua, Zhejiang, China; ^2^Department of Obstetrics and Gynecology, Affiliated Jinhua Hospital, Zhejiang University School of Medicine, Jinhua, Zhejiang, China; ^3^Taizhou Medical College, Taizhou, Zhejiang, China

**Keywords:** vitamin D, pregnant women, deficiency odds, gestational weeks, non-linear change

## Abstract

**Background:**

There is an incomplete understanding of fluctuations in vitamin D (VitD) concentration during pregnancy among Chinese women. Furthermore, previous research has yielded conflicting results in this area. This study aims to investigate the changes in VitD status and deficiency in Chinese pregnant women across various age groups, gestational weeks, and as well as seasonal variations through conducting a large-scale survey.

**Methods:**

A toal of 11,220 Chinese pregnant women between 2021 and April 2023 were included in this study. Generalized additive models (GAM), stratified analysis, and restricted cubic splines (RCS) were used to analyze changes in VitD status and deficiency risk during pregnancy.

**Results:**

Of the participants, 45.2% had deficient concentration of 25-hydroxyvitamin D. VitD concentration and deficiency rate do not show linear changes with age and gestational weeks. With increasing gestational weeks, VitD concentration rapidly increased in women with gestational age < 20 weeks, remained stable between 20 and 30 weeks, and decreased beyond 30 weeks; however, the odds of VitD deficiency showed three different patterns: a rapid decline, a stable period, and a mild increase, respectively. Based on the stratified regression analysis, VitD deficiency odds increased by 16% with each additional week of gestation in pregnant women with gestational age > 30 weeks, OR = 1.16 (1.10–1.22), *p* < 0.001. Interaction effect analysis indicated that pregnant women over 35 years with gestational weeks between 20 and 30 had the lowest odds of VitD deficiency.

**Conclusion:**

VitD concentration undergo three phases during pregnancy: rapid increase, plateau, and subsequent decrease. VitD deficiency odds was highest in pregnant women under 25 with gestational ages <20 and lowest in pregnant women over 35 with gestational ages between 20 and 30. The odds of deficiency increase slightly in pregnant women with gestational ages beyond 30 weeks, indicating that they may require additional VitD supplementation.

## 1. Introduction

Vitamin D is a secosteroid with an endocrine mechanism of action which is sequentially synthesized in humans in the skin, liver and kidneys. The active hormone, 1alpha,25-dihydrocholecalciferol [1,25(OH)2D3], is often considered only in terms of its role in controlling calcium and phosphorus homeostasis (1, 2). Apart from this, VitD is also involved in immune regulation, nerve transmission, and cell differentiation, and has been linked to the prevention and treatment of multiple diseases (1–4). The Chinese dietary guidelines (2022 edition) recommend that pregnant women should aim for 10–20 min of sunlight exposure per day, on average; pregnant women living in high latitude areas, experiencing sunlight deficiency during winter, or having insufficient outdoor activities to synthesize VitD through sunlight should consider taking VitD supplements, with a dosage of 10 μg/day (400 IU/day) (5). The deficiency of VitD during pregnancy can lead to adverse consequences for both the mother and the fetus (6). Numerous studies have highlighted that pregnant women with low concentrations of VitD are associated with negative outcomes, such as low birth weight, skeletal deformities, and impaired cognitive development in infants (6–8). Furthermore, some studies have also shown that VitD deficiency during pregnancy is associated with an increased risk of pregnancy complications, such as gestational hypertension, gestational diabetes, and postpartum maternal health issues, such as postpartum depression (9, 10).

VitD deficiency in pregnant women is very common worldwide. Ginde et al. (11) reported that 46, 32, and 18% of pregnant women suffered from VitD deficiency during the first (T1), second (T2), and third trimesters (T3) of pregnancy in America, respectively. In a study conducted on 125 Chinese healthy pregnant women, Song et al. found that the prevalence of VitD deficiency was 96.8% (12). Xiang et al. recruited 311 pregnant women and indicated that VitD deficiency, insufficiency, and sufficiency were found in 260 (83.6%), 39 (12.5%), and 12 (3.9%) women, respectively (13). However, these Chinese studies had used small samples, which might result in sampling bias. Moreover, there are differences in research results regarding the timing and rate of VitD deficiency during pregnancy (14, 15). A study conducted in southern China indicated that the 25-hydroxyvitamin D [25(OH)D] concentration increased with gestational week (14). However, Kovacs found that VitD concentration undergo three phases, including increase, plateau, and decrease, during the course of pregnancy (15). Therefore, it is necessary to gain a deeper understanding of the incidence and changes of VitD deficiency during pregnancy in Chinese women. This study aims to evaluate the prevalence of VitD deficiency in pregnant women in southern China and its changes during pregnancy, in order to better understand the status of VitD deficiency in pregnant women and to provide scientific and effective prevention and intervention measures for maternal health.

## 2. Materials and methods

### 2.1. Study population

This retrospective, large-scale study was based on pregnant women who attended pregnancy tests at Affiliated Jinhua Hospital, Zhejiang University School of Medicine and Jinhua Maternal and Child Health Care Hospital from 2021 to April 2023. Jinhua is located in the central part of Zhejiang Province, China, with geographical coordinates of 29.08°N latitude and 119.65°E longitude. It belongs to the subtropical monsoon climate zone, with hot and humid summers, cold and dry winters, and warm and pleasant springs and autumns. The average annual sunshine time in Jinhua is about 1,800 h, with good solar radiation resources. In the present study, the inclusion criteria were: (1) pregnant women with age ≥ 18 years old; (2) gestational age over 5 weeks; (3) no family history of mental disease; (4) no serious trauma during pregnancy, such as broken bones. The exclusion criteria of pregnant women were: (1) any mental illness prior to pregnancy; (2) premature miscarriage; (3) abnormal fetal development or fetal anomalies; (4) serious chronic diseases, such as hypertension, diabetes, cardiovascular disease, chronic kidney disease, autoimmune disease and among others. It is worth noting that, in the absence of obvious calcium deficiency symptoms, pregnant women participating in prenatal examination in these two hospital were usually advised by the obstetricians to regularly supplement with VitD (400 IU/day) starting at 20 weeks of gestation. Finally, 11,220 pregnant women with an average age of 30 ± 5 years old were included in this study. The proportion of the participants aged less than 25 years was 14.49, and 15.62% of the subjects were over 35 years old. This study only included the results of the first VitD testing of pregnant women in these two hospitals, as the results of the second testing might be influenced by their additional VitD supplement dosage according to the first low VitD concentrations. The study approved by the Ethics Committee of the Affiliated Jinhua Hospital, Zhejiang University School of Medicine, approval No. (199) of Ethical Review Committee, May 9th, 2023. Patients and the public were not involved in the design, or conduct, or reporting, or dissemination plans of our research. According to the regulations of the Ethics Committee of the Affiliated Jinhua Hospital, Zhejiang University School of Medicine, the consent for participation is not necessary for this this retrospective study.

### 2.2. The basic characteristics and measurement of serum VitD

All the basic clinical characteristics were obtained from the electronic medical record system, including age, week of pregnancy, sampling date, the concentration of serum 25(OH)D. In the present study, serum 25(OH)D concentration was used to represent VitD status of the pregnant women. The concentrations of 25(OH)D_2_ and 25(OH)D_3_ were obtained from fasting blood samples of the pregnant women by liquid chromatography–tandem mass spectrometry, and the total serum concentration of 25(OH)D was the sum of 25(OH)D_2_ and 25(OH)D_3_. The lowest limits of detection were 2 ng/mL for 25(OH)D_2_ and 5 ng/mL for 25(OH)D_3_. Samples with concentrations below these limits were recorded as 2 ng/mL for 25(OH)D2 and 5 ng/mL for 25(OH)D3, respectively.

### 2.3. Definition of VitD deficiency

According to the Chinese Health Industry Standard WS/T 677–2020: method for VitD deficiency screening (16), 25(OH)D concentration between 12 and 20 ng/mL was defined as insufficiency, 25(OH)D concentration less than 12 ng/mL was defined as deficiency. In the present study, VitD deficiency was defined as a 25(OH)D concentration less than 20 ng/mL, with VitD concentration ranging from ≥12 to <20 ng/mL defined as mild deficiency, and concentration < 12 ng/mL defined as severe deficiency.

### 2.4. Definitions of subgroups

In the present study, all the 11,220 participants were divided into subgroups according to gestational week, sampling season and age group. The 1st–12th, 13th–27th, after 28th week of gestation were defined as the first (T1), second (T2) and third trimester (T3), respectively. Similarly, the participants were divided into three age groups: age group 1 (< 25 years), age group 2 (25–34 years old), and age group 3 (over 35 years old). The sampling period was divided into four seasons: spring (March–May), summer (June–August), autumn (September–November), and winter (December–February). Additionally, based on the generalized additive models (GAM) and restricted cubic splines (RCS) analysis, VitD concentration during gestation exhibited three distinct stages in relation to gestational week: stages of an increase at <20 weeks of gestation, a plateau between 20 and 30 weeks, and a decrease after 30 weeks of gestation. Accordingly, gestational weeks were re-categorized into three groups: Gestational stage A (< 20 weeks), Gestational stage B (20–30 weeks), and Gestational stage C (> 30 weeks).

### 2.5. Statistical analysis

All the statistical analyses in the present study were conducted by using SPSS 26.0 statistical software, R software (4.2.3 version). Variables that followed a normal distribution were reported as mean ± standard deviation (SD), and categorical variables were presented as numbers and percentages (%). Unpaired t-tests, and ANOVA were performed to execute the between-group comparisons of measurement data. Pearson’s chi-squared tests was used to analyze the differences between the groups of count data. GAM was used to analyze the association of VitD concentration with gestational weeks and age of the pregnant women. RCS analysis was performed to examine the relationships between VitD deficiency odds, gestational weeks and age of the pregnant women. The relationships of VitD deficiency odds with age and gestational weeks in pregnant women across different age groups, gestational weeks, and seasons was performed by using stratified analysis based on logistics regression. A two-tailed value of *p* <0.05 was considered statistically significant in this study.

## 3. Results

### 3.1. The VitD status in three trimesters during pregnancy

**Figure 1:**
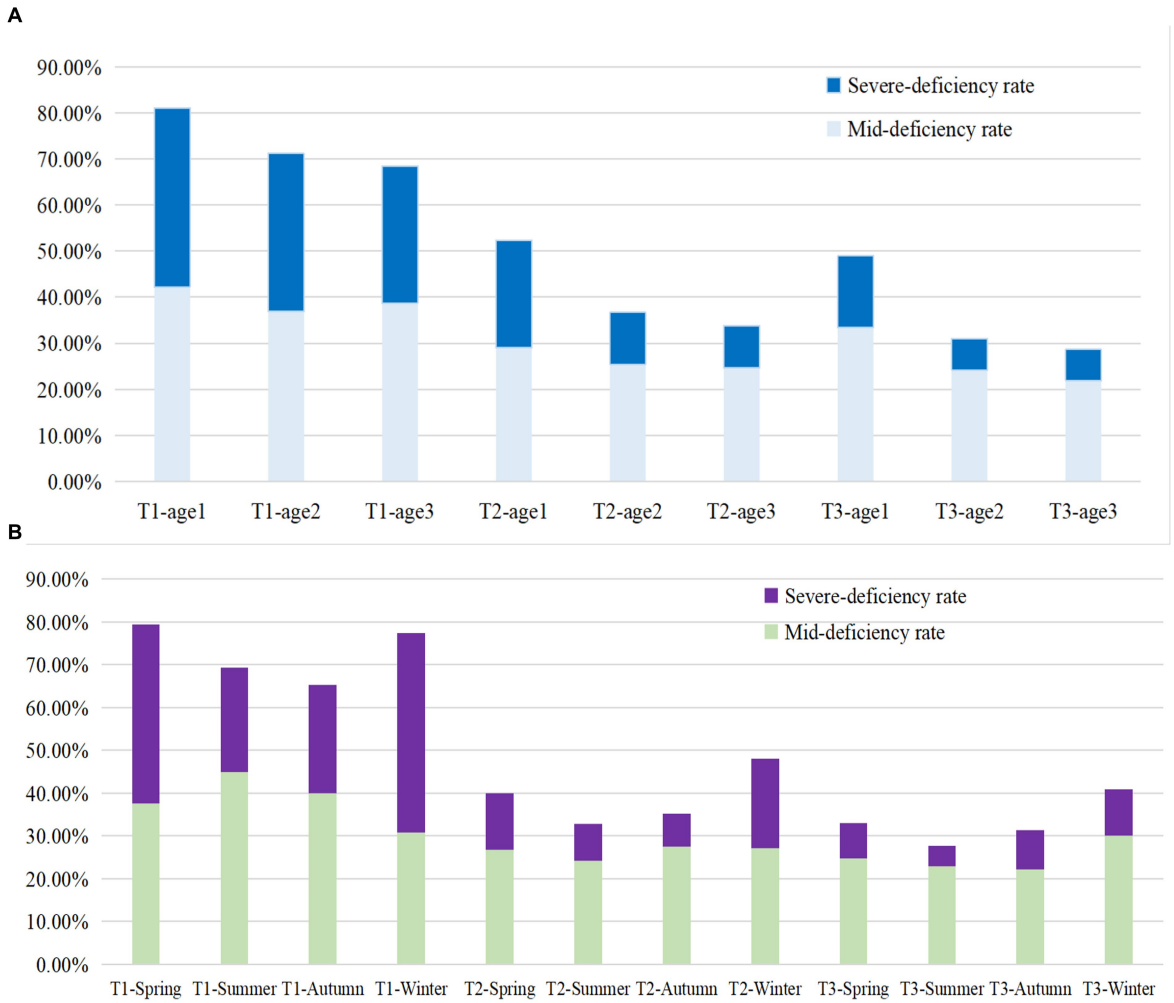
The VitD deficiency rate in different Age groups **(A)** and sampling seasons **(B)** among pregnant women during three trimesters. Age group 1: <25 years old; Age group 2: 25–34 years old; Age group 3: ≥ 35 years old; T1: 1st–12th week of gestation, T2: 13th–27th week of gestation, T3: after 28th week of gestation. Mid-deficiency: VitD concentration ranging from ≥12 to <20 ng/mL; Severe deficiency: VitD concentration < 12 ng/mL.

This study included a total of 11,220 Chinese pregnant women, whose average VitD concentration was 23 ± 11 ng/mL, and the VitD deficiency rate was 45.2%. Of them, 2,736, 5,371, and 3,113 were in the T1, T2 and T3 of pregnancy, respectively. Overall, the average concentration of VitD increased gradually from T1 to T3. Correspondingly, the rates of VitD deficiency decreased gradually in T1, T2, and T3, being 72.33, 38.65, and 32.86%, respectively. The VitD concentration and the VitD deficiency rate in pregnant women during three trimesters were shown in Table 1 and Figure 1. Regardless of age group or season, there was always an increasing trend (*p* < 0.001) in the average VitD concentration from the T1 to T2 to T3 periods, while the prevalence of VitD deficiency showed a decreasing trend (*p* < 0.001). Moreover, there was an age-related increase in the average VitD concentration and a decrease in the rate of VitD deficiency observed among pregnant women during both T1 and T2 periods. The concentrations of VitD collected during the Summer and Autumn were higher than those collected in the Winter and Spring, with the greatest difference observed among pregnant women during the T1 period (*p* < 0.001), however, the deficiency rate of VitD was found to be the opposite. It is noteworthy that even within the same VitD status (normal, mild deficiency, or severe deficiency), the VitD concentrations were found to be lowest among pregnant women during the T1 period compared to those during the T2 and T3 periods (*p* < 0.001).

**Table 1:** The comparison of VitD concentrations in pregnant women during three trimesters between different groups.

Subgroups	The VitD concentrations (ng/mL)			
	T1 (*n* = 2,736)	T2 (*n* = 5,371)	T3 (*n* = 3,113)	*p-*value
Total	16 ± 7	24 ± 10	26 ± 11	<0.001
Age, years				
<25 years old	15 ± 6	21 ± 10	22 ± 10	<0.001
25–34 years old	16 ± 8	25 ± 11	27 ± 11	<0.001
≥35 years old	17 ± 8	25 ± 10	27 ± 10	<0.001
*p*-value	<0.001	<0.001	<0.001	
Sampling season				
Spring	15 ± 7	24 ± 11	27 ± 12	<0.001
Summer	17 ± 7	25 ± 10	27 ± 10	<0.001
Autumn	18 ± 7	25 ± 10	26 ± 10	<0.001
Winter	15 ± 8	23 ± 11	25 ± 12	<0.001
*p*-value	<0.001	<0.001	<0.001	

VitD, vitamin D; ANOVA analyses was applied for between group comparison. T1, 1st–12th week of gestation, T2, 13–27th week of gestation, T3, after 28th week of gestation.

### 3.2. Associations of VitD concentrations with gestational week and age based on GAM analysis

**Figure 2:**
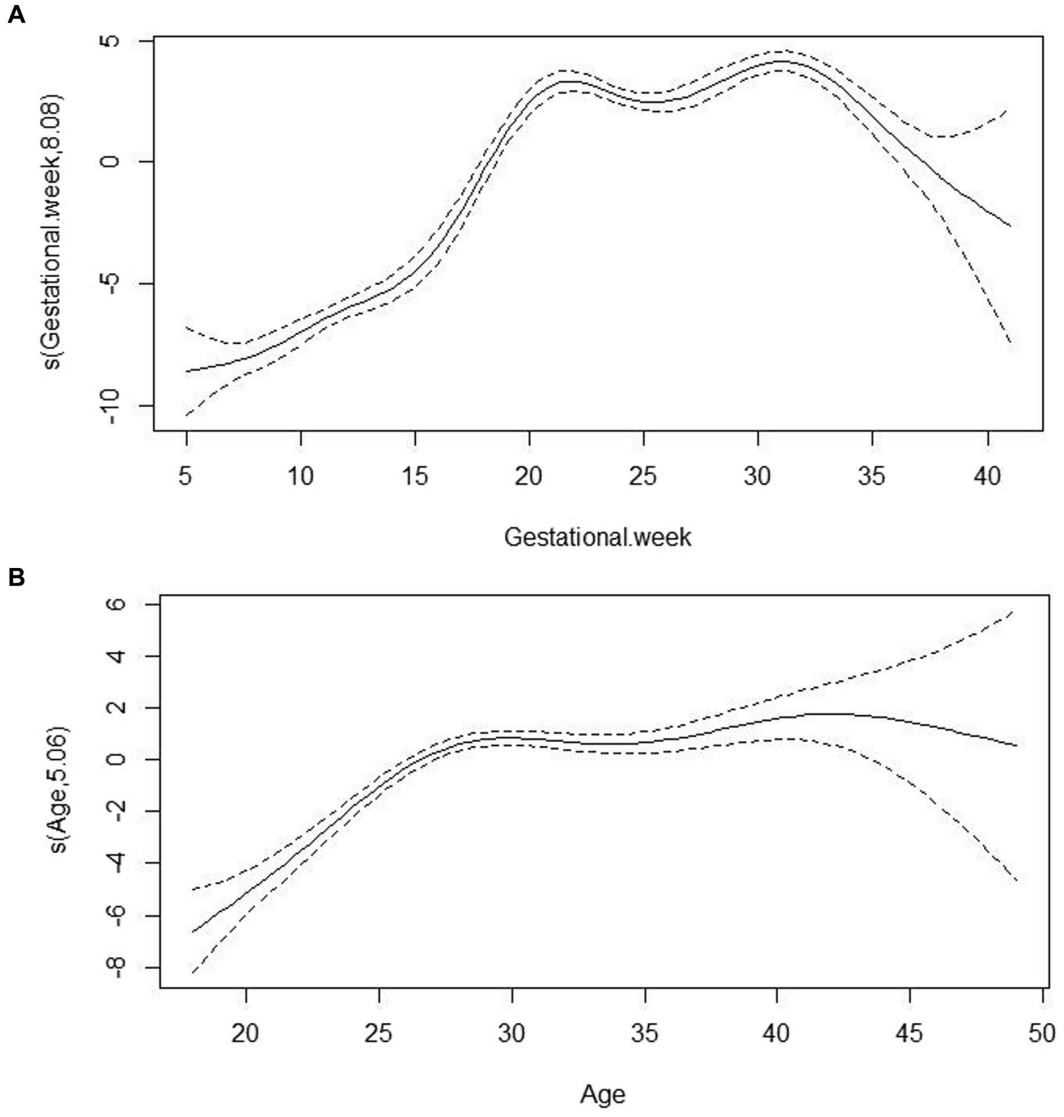
The associations of VitD concentrations with gestational week **(A)** and age **(B)** based on Generalized additive models analysis. s(Gestational week, 8.08) represented the smoothed value obtained by smoothing the relationship between gestational week and VitD concentration in the model, with gestational week as the independent variable; in this model, the corresponding impact or trend of gestational week on VitD concentration was estimated to be 8.08 after the smoothing process. s(Age, 5.06) represented the corresponding impact or trend of age on VitD concentration was estimated to be 5.06 after the smoothing process.

After adjusting for the confounding factors of age and sampling season, the relationship between gestational week and VitD concentrations is shown in Figure 2A. Overall, with increasing weeks of gestation, VitD concentrations showed a rapid increase at <20 weeks of gestation, a relatively stable and high concentration between 20 and 30 weeks of gestation, and a decrease after >30 weeks of gestation. Figure 2B showed the relationship between age and VitD concentration after adjusting for the confounding factors of sampling season and gestational week. In general, the relationship between VitD concentrations and age showed a curve that increased initially and then became relatively stable. Compared to the changes in VitD concentrations with increasing gestational age, the changes in VitD concentrations with increasing age were relatively less pronounced.

### 3.3. Associations of VitD deficiency odds with gestational week and age based on RCS analysis

**Figure 3:**
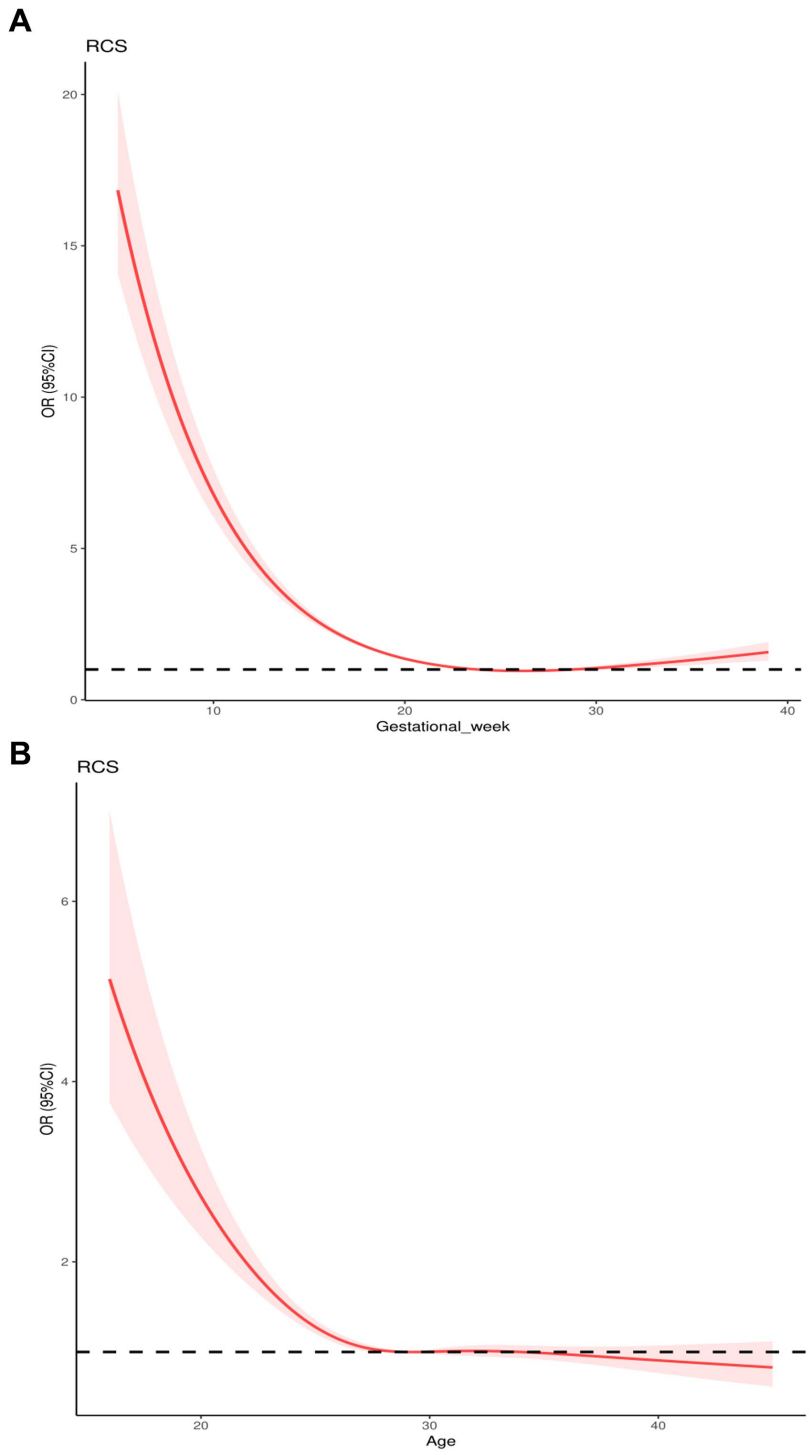
The associations of VitD deficiency odds with gestational week **(A)** and age **(B)** based on restricted cubic splines analysis analysis.

Figures 3A,B demonstrated the nonlinear associations of VitD deficiency odds with gestational week (p for non-linear <0.001) and age (p for non-linear <0.001) after adjustment for the potential confounders in Chinese pregnant women performed by using RCS analysis, respectively. During pregnancy, the odds of VitD deficiency experienced a rapid decline as gestational weeks progress before 20 weeks. Between 20 and 30 gestational weeks, the odds of VitD deficiency maintained a relatively low concentration. However, in pregnant women >30 gestational weeks, the odds of VitD deficiency slightly increased with the progression of gestational weeks. However, after adjusting for confounding factors such as gestational week and season, the relationship between age and the odds of VitD deficiency demonstrated an initial rapid decline in VitD deficiency rates with increasing age, followed by a tendency to maintain at a lower concentration.

### 3.4. Stratified analysis based on logistic regression

**Figure 4:**
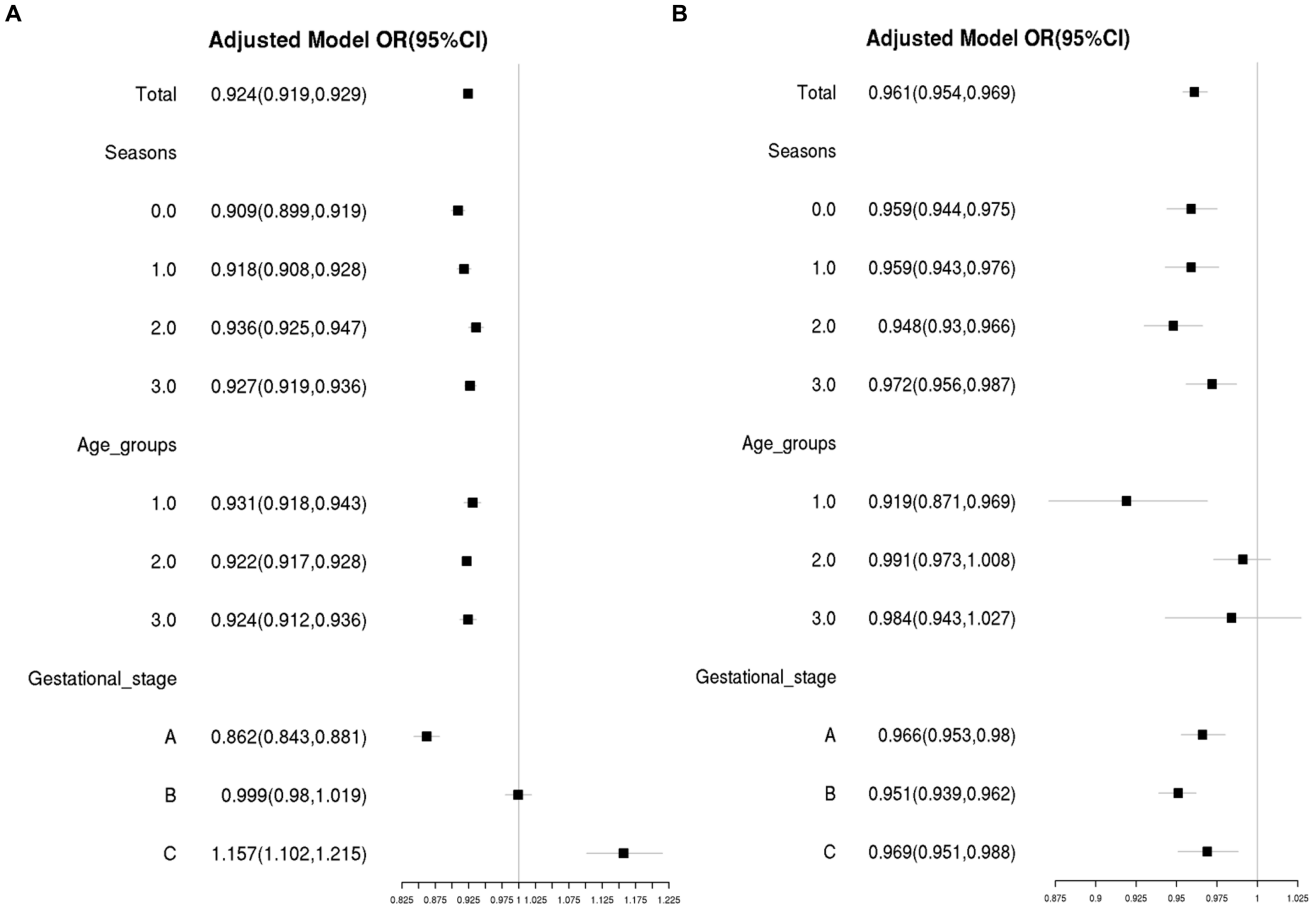
The associations of VitD deficiency odds with gestational week **(A)** and age **(B)** in different subgroups. The confounding factors included age and gestational week, as appropriate. Season 0, 1, 2, and 3 were Spring, Summer, Autumn and Winter, respectively. Age group 1: <25 years old; Age group 2: 25–34 years old; Age group 3: ≥ 35 years old; Gestational stage A: <20 weeks of gestation; Gestational stage B: 20–30 weeks of gestation; Gestational stage C: >30 weeks of gestation.

Based on the relationship between gestational week and VitD status analyzed using GAM and RCS, gestational weeks were re-categorized into three groups: Gestational stage A (< 20 weeks), Gestational stage B (20–30 weeks), and Gestational stage C (> 30 weeks). The associations of VitD deficiency odds with gestational week and age in different groups were evaluated by stratified analysis. As shown in Figure 4A, in four sampling seasons, three Age groups and Gestational stage A, the odds of VitD deficiency significantly decreased with increasing gestational age (OR < 1, *p* < 0.05), respectively. However, among the pregnant women with Gestational stage C, the VitD deficiency odds increased by 16% with each additional week of gestation, OR = 1.16 (1.10–1.22), *p* < 0.001. Overall, the odds of VitD deficiency decreased with increasing age in each subgroup (OR < 1), respectively, even though the changes were not statistically significant among Age group 2 and Age group 3 (Figure 4B). Additionally, Table 2 listed the interaction effects of age and gestational week for VitD deficiency odds. The interaction effect analysis indicated that after adjustment for sampling season, the pregnant women over 35 years of age with gestational age between 20–30 weeks had the lowest VitD deficiency odds compared to other groups, OR = 0.21 (0.18–0.25), *p* < 0.001.

**Table 2:** The association of VitD deficiency with season, gestational stage and age.

	Crude OR	*p-*value	Adjusted OR^#^	*p-*value
Sampling season				
Spring	Reference	–	Reference	–
Summer	0.76 (0.68–0.84)	<0.001	0.71 (0.63–0.79)	<0.001
Autumn	0.8 (0.71–0.89)	<0.001	0.76 (0.67–0.85)	<0.001
Winter	1.31 (1.18–1.45)	<0.001	1.28 (1.15–1.43)	<0.001
Gestational stage				
<20 weeks of gestation	Reference	–	Reference	–
20–30 weeks of gestation	0.23 (0.21–0.26)	<0.001	0.23 (0.21–0.25)	<0.001
>30 weeks of gestation	0.25 (0.22–0.28)	<0.001	0.24 (0.21–0.27)	<0.001
Age group				
<25 years old	Reference	–	Reference	–
25–34 years old	0.54 (0.49–0.6)	<0.001	0.52 (0.46–0.59)	<0.001
≥ 35 years old	0.46 (0.4–0.53)	<0.001	0.46 (0.4–0.54)	<0.001
Interaction effect of age and gestational stages				
Age group 1 and Gestational stage A	Reference	–	Reference	–
Age group 2 and Gestational stage B	0.26 (0.24–0.29)	<0.001	0.25 (0.23–0.28)	<0.001
Age group 2 and Gestational stage C	0.27 (0.24–0.31)	<0.001	0.26 (0.23–0.29)	<0.001
Age group 3 and Gestational stage B	0.21 (0.18–0.25)	<0.001	0.21 (0.18–0.25)	<0.001
Age group 3 and Gestational stage C	0.26 (0.21–0.33)	<0.001	0.25 (0.19–0.32)	<0.001

^#^The confounding factors included sampling season, age and gestational week, as appropriate. VitD: vitamin D; Age group 1: <25 years old; Age group 2: 25–34 years old; Age group 3: ≥ 35 years old; Gestational stage A: <20 weeks of gestation; Gestational stage B: 20–30 weeks of gestation; Gestational stage C: >30 weeks of gestation.

## 4. Discussion

In the present study, we found that the prevalence of VitD deficiency among Chinese pregnant women, particularly those who were young and in early pregnancy, and sampled during winter. Moreover, the concentrations and prevalence of VitD deficiency did not show a linear trend with increasing age and/or gestational weeks. In particular, VitD concentrations in pregnant women showed different patterns with increasing gestational weeks depending on the gestational age. Specifically, in women with gestational age less than 20 weeks, VitD concentrations increased rapidly with gestational weeks, remained relatively stable in women with gestational age between 20 and 30 weeks, and decreased with increasing gestational weeks in women with gestational age greater than 30 weeks. On the other hand, the VitD deficiency rate showed an opposite trend, with a rapid decline, a stable period and a mild increase were observed in women with gestational age less than 20 weeks, between 20 and 30 weeks, and greater than 30 weeks, respectively. Interestingly, in pregnant women with a gestational age greater than 30 weeks, the probability of VitD deficiency increases by approximately 16% with each passing week of gestational age. Finally, we found that after adjusting for seasonal factors, pregnant women aged over 35 with a gestational age between 20 and 30 weeks had the lowest probability of VitD deficiency; conversely, pregnant women under the age of 25 with a gestational age less than 20 weeks had the highest probability of VitD deficiency.

VitD plays an important role in maintaining the bone health and overall health of pregnant women. Studies have shown that VitD deficiency during pregnancy may increase the risk of adverse pregnancy outcomes such as pre-eclampsia, gestational diabetes, low birth weight, and preterm birth (17–23). The study conducted by Lu et al. revealed that vitamin D deficiency significantly increased the risk of gestational diabetes mellitus by 45% (21). A prospective cohort study discovered that the VitD concentration was significantly lower in patients diagnosed with pre-eclampsia, in comparison to normal pregnant women and the risk of developing pre-eclampsia was 2.48 (95% confidence interval, 1.51–4.08) times higher in the hypovitaminosis D group than in the VitD adequate group (22). A cross-sectional study conducted in China demonstrated that low VitD concentrations in pregnant women increased the risk of spontaneous abortion by 1.71-fold (23). For pregnant women, VitD concentration is closely related to inadequate intake of VitD in their diet, however, due to the low content of VitD in food sources and factors such as food types, seasons, and processing methods, it poses significant challenges for pregnant women to obtain sufficient VitD through diet (14). Some studies and guidelines have emphasized the special status of pregnant women in terms of VitD requirements. VitD supplementation can effectively meet the additional needs of pregnant women, and positively impact their health through various physiological processes such as regulating calcium and phosphate absorption, participating in bone formation and skeletal health, and modulating immune function (20, 24, 25). The importance of VitD supplementation during pregnancy in preventing some of the health risks to the mother and fetus appears linked to achieving 25(OH)D concentrations >40 ng/mL, the beginning point of the plateau where conversion of the VitD metabolite 25(OH)D, the pre-hormone, to 1,25(OH)2D, the active hormone, is optimized (26). However, VitD deficiency is common among pregnant women worldwide, the prevalence of VitD during pregnancy was reported to range from 32 to 74% across different countries (27–29). So far, the understanding of fluctuations in VitD status in Chinese women during pregnancy is still not comprehensive or limited. This study aims to investigate the changes of VitD status and deficiency risk during pregnancy among Chinese pregnant women of different age groups, gestational weeks, and seasons by conducting a large-scale survey.

The present study showed a high rate of VitD deficiency in Chinese pregnant women, with deficiency rates of 72.33, 38.65, and 32.86% in T1, T2, and T3, respectively; furthermore, the VitD concentrations increased rapidly before 20 weeks of gestation. These findings were similar to a previous study (14). However, the study involving 4,368 Chinese pregnant women revealed that VitD concentrations gradually increased with gestational weeks during pregnancy (14); in contract, our present study observed a different pattern, where VitD concentrations remained relatively stable in pregnant women between 20 and 30 weeks after a rapid increase in the first 20 weeks, but displayed a decreasing trend in pregnant women beyond 30 weeks. A previous study from the UK also indicated a non-linear tendency for the VitD status to increase with the progression of pregnancy, with a decline in VitD deficiency rate in early pregnancy followed by an increase in late pregnancy (30). Although the VitD deficiency rate of pregnant women in the UK study did not experience a period of relative stability, the result of an increase in deficiency rate during late pregnancy was consistent with the findings of the present study.

In this study, the concentration of VitD in pregnant women less than 20 weeks of gestation gradually increased, remained stable at a relatively high concentration between weeks 20 and 30, and decreased with increasing gestational weeks in women with gestational age greater than 30 weeks. It maybe due to the following reasons: (1) although the demand for VitD increased during pregnancy, the required amount had not yet reached a very high concentration in early pregnancy, and the absorption and metabolism capacity of VitD in pregnant women gradually increases in the early stages of pregnancy (31, 32). (2) Due to local prenatal care recommendations, pregnant women were advised to start regular VitD supplements around week 20 of pregnancy. (3) In pregnant women beyond 30 gestational weeks, the increased demand for VitD by the fetus in the later stages of pregnancy and the reduced ability of pregnant women to metabolize VitD, coupled with factors such as season and outdoor activities, leading to the decrease of VitD concentration (33–35).

In addition to factors such as gestational week and season, there was a strong relationship between maternal age and VitD deficiency. Many previous studies had shown that age was an important factor affecting VitD concentrations in pregnant women, and that older pregnant women tended to have lower concentrations of VitD and higher prevalence of VitD deficiency (36, 37). However, in the present study, the VitD concentrations and deficiency rate among the pregnant women over 35 years old were significantly higher and lower than those among the pregnant women under 25 years old, respectively. This finding was similar to another study conducted on Chinese pregnant women, which indicated that the risk of VitD deficiency in pregnant women over 30 years old was only 68% of that in pregnant women under 25 years old (29). Due to differences in geographical environment, dietary habits, and other factors among study populations, the relationship between age and VitD status had produced varying results in different studies (29, 36, 37). Moreover, older pregnant women might have more experience regarding diet and lifestyle habits during pregnancy, which could contribute to a lower prevalence of VitD deficiency compared to younger pregnant women.

The highlights of the present study were that we revealed the three-stage pattern of rapid increase, plateau, and decline in VitD concentrations during pregnancy among Chinese women; moreover, we also elaborated on the relationship between VitD deficiency and gestational age in pregnant women over 30 weeks, demonstrating that as gestational age increased by 1 week, the odds of VitD deficiency increased by 16% in this population. However, the major limitation of this study was the lack of information on the dietary intake and supplementation of VitD, as well as the economic status and occupation of the pregnant women. Despite all pregnant women in this study were advised by their antenatal care providers to start taking VitD supplements around their 20th week of pregnancy, it was difficult to evaluate the compliance of pregnant women without obvious clinical symptoms, as well as their frequency of supplementation and consumption of additional VitD-rich foods.

## 5. Conclusion

In conclusion, this study revealed a non-linear trend in the concentrations and deficiency rates of VitD in Chinese pregnant women, as well as complex relationships with factors such as age, gestational age, and season. These findings suggest that VitD supplementation should start early in pregnancy, rather than the current recommendation by local prenatal doctors to begin at 20 weeks, furthermore, it is recommended to monitor VitD concentrations during pregnancy and increase VitD supplementation as needed among pregnant women after 30 weeks of gestation, particularly for the young pregnant women in China. This study provides new ideas and directions for monitoring and intervention of VitD in pregnant women. We believe that strengthening health education on VitD for pregnant women, and improving monitoring and supplementation systems for VitD in pregnant women can help reduce VitD deficiency rates. However, it is also important to note the limitations of this study, such as the need for more comprehensive data and experimental designs to explore the more complex relationships between VitD deficiency and health in pregnant women.

## Data availability statement

The raw data supporting the conclusions of this article will be made available by the authors, without undue reservation.

## Ethics statement

The studies involving humans were approved by the Ethics Committee of the Affiliated Jinhua Hospital, Zhejiang University School of Medicine. The studies were conducted in accordance with the local legislation and institutional requirements. Written informed consent for participation was not required from the participants or the participants’ legal guardians/next of kin because according to the regulations of the Ethics Committee of the Affiliated Jinhua Hospital, Zhejiang University School of Medicine, the consent for participation is not necessary for this retrospective cross-sectional study.

## Author contributions

HW and YM: study design. HW, YM, and MF: statistical analysis. XS and HW: manuscript writing. XS, HW, and BL: data collection. FZ, HW, MF, and BL: manuscript revision. All authors contributed to the article and approved the submitted version.
